# Exploring the Diversity and Specificity of Secondary Biosynthetic Potential in *Rhodococcus*

**DOI:** 10.3390/md22090409

**Published:** 2024-09-06

**Authors:** Gang-Ao Hu, Yue Song, Shi-Yi Liu, Wen-Chao Yu, Yan-Lei Yu, Jian-Wei Chen, Hong Wang, Bin Wei

**Affiliations:** 1 College of Pharmaceutical Science & Collaborative Innovation Center of Yangtze River Delta Region Green Pharmaceuticals, Zhejiang Key Laboratory of Green, Low-Carbon, and Efficient Development of Marine Fishery Resources, Zhejiang University of Technology (ZJUT), Hangzhou 310014, China; 201706030718@zjut.edu.cn (G.-A.H.); 2112107104@zjut.edu.cn (Y.S.); 201906030311@zjut.edu.cn (S.-Y.L.); 2112007282@zjut.edu.cn (W.-C.Y.); yanleiyu@zjut.edu.cn (Y.-L.Y.); cjw983617@zjut.edu.cn (J.-W.C.); 2 Binjiang Institute of Artificial Intelligence, Zhejiang University of Technology (ZJUT), Hangzhou 310051, China

**Keywords:** *Rhodococcus*, biosynthetic gene clusters, comparative genomics, high-throughput elicitor screening, non-targeted metabolomics

## Abstract

The actinomycete genus *Rhodococcus* is known for its diverse biosynthetic enzymes, with potential in pollutant degradation, chemical biocatalysis, and natural product exploration. Comparative genomics have analyzed the distribution patterns of non-ribosomal peptide synthetases (NRPSs) in *Rhodococcus*. The diversity and specificity of its secondary metabolism offer valuable insights for exploring natural products, yet remain understudied. In the present study, we analyzed the distribution patterns of biosynthetic gene clusters (BGCs) in the most comprehensive *Rhodococcus* genome data to date. The results show that 86.5% of the gene cluster families (GCFs) are only distributed in a specific phylogenomic-clade of *Rhodococcus*, with the most predominant types of gene clusters being NRPS and ribosomally synthesized and post-translationally modified peptides (RiPPs). In-depth mining of RiPP gene clusters revealed that *Rhodococcus* encodes many clade-specific novel RiPPs, with thirteen core peptides showing antibacterial potential. High-throughput elicitor screening (HiTES) and non-targeted metabolomics revealed that a marine-derived *Rhodococcus* strain produces a large number of new aurachin-like compounds when exposed to specific elicitors. The present study highlights the diversity and specificity of secondary biosynthetic potential in *Rhodococcus*, and provides valuable information for the targeted exploration of novel natural products from *Rhodococcus*, especially for phylogenomic-clade-specific metabolites.

## 1. Introduction

Actinobacteria is the major source of natural products in bacteria, with well-known genera such as *Streptomyces*, *Micromonospora*, *Nocardia*, and *Saccharothrix* [1,2]. The genus *Rhodococcus* was first proposed by Zopf in 1891 and contains 55 species with validly published and correct names nowadays [3]. *Rhodococcus* has received widespread attention for its pivotal role in degrading a wide range of natural and xenobiotic compounds, but has received less attention in the discovery of natural products [4]. Currently, only 24 secondary metabolites have been reported to be derived from *Rhodococcus*, including rhodopeptins [5], lariatins [6], aurachins [7], rhodostreptomycin [8], rhodochelin [9], and saframycin [10]; however, these compounds showed excellent and diverse biological activities, such as antibacterial, antifungal, antitrypanosomal, anticancer, and siderophores [4].

McLeod et al. sequenced the complete genome of the first *Rhodococcus* strain (*Rhodococcus jostii* RHA1), and found that the strain encodes up to 24 nonribosomal peptide synthetases (NRPSs) and 7 polyketide synthases (PKSs) [11]. In addition, two recent large-scale genome mining studies have revealed that *Rhodococcus* encodes numerous novel biosynthetic gene clusters (BGCs) for secondary metabolites, highlighting its significant potential in producing novel natural products [12,13]. However, more than 1000 genomes of *Rhodococcus* have been publicly released, and it is crucial to choose the appropriate *Rhodococcus* strain for the exploration of novel secondary metabolites. Our previous research has shown that the distribution patterns of BGCs encoded by multiple genera, such as *Bacillus*, *Allokutzneria*, and *Kibdelosporangium*, exhibit species or genus specificity [14,15]. Furthermore, in-depth exploration of these genomes can help in the targeted discovery of specific types of natural products.

*Rhodococcus* has been isolated from a wide range of environments, including marine, aquatic, soil, animals, plants, and insects [16]. Based on the study by Agustina Undabarrena et al., phylogenomic analysis of 110 *Rhodococcus* strains indicates that the distribution of *Rhodococcus* species in the evolutionary tree correlates to some extent with their isolation sources. However, eight *Rhodococcus* strains from marine sources are randomly distributed across all four clades of the evolutionary tree [13]. A marine-derived strain, *Rhodococcus* sp. H-CA8f was found to possess a unique BGC distribution within its phylogenomic clade [13]. These findings highlight that *Rhodococcus* strains from marine sources possess greater genetic diversity and more unique secondary metabolite potential.

Therefore, in the present study, we conducted a systematic analysis of the distribution patterns of BGCs and gene cluster families (GCFs) within the most comprehensive *Rhodococcus* genome dataset to date, which were classified into distinct phylogenomic clades. While Undabarrena et al. [13] primarily focused on an in-depth analysis of nonribosomal peptide synthetase (NRPS) GCFs and highlighted the phylogenomic-dependent patterns of NRPS GCFs in *Rhodococcus*, our research expands on this by analyzing the clade-specific distribution of all major BGC types—eight in total. This comprehensive approach provides a broader understanding of the biosynthetic potential within the *Rhodococcus* genus, extending beyond just NRPS clusters. Additionally, we conducted a detailed analysis of the composition of ribosomally synthesized and post-translationally modified peptide (RiPP) GCFs and the metabolite scaffolds they encode. By utilizing deep-learning algorithms, we rapidly identified dozens of novel antimicrobial peptides from the core peptides encoded by RiPP gene clusters. Furthermore, we explored the secondary metabolites of a marine-derived *Rhodococcus* strain through a combined strategy of high-throughput elicitor screening (HiTES) [17] and non-targeted metabolomics. This approach led to intriguing discoveries, including the production of a series of aurachin-like compounds, which have not been previously reported.

## 2. Results

### 2.1. The Overall Distribution of BGCs in Rhodococcus

**Figure 1:**
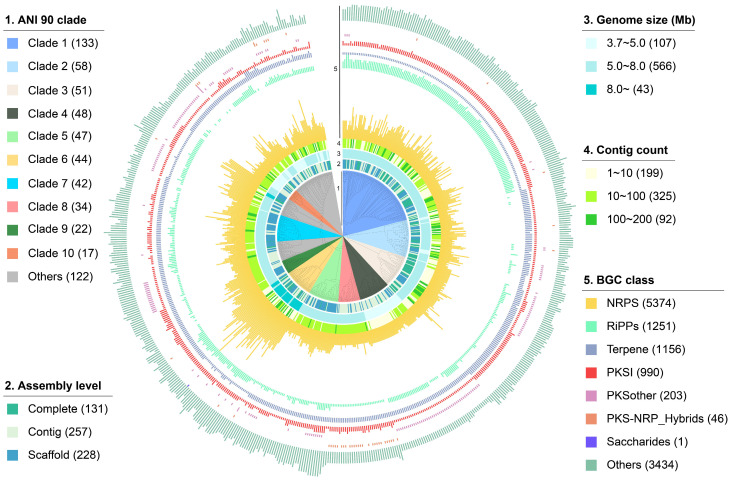
Heatmap and hierarchical clustering based on pairwise ANI values of 616 *Rhodococcus* genomes. Representation of the data (inner to outer layers): phylogenomic clade, assembly level, genome size, contig count, and the numbers of each class of BGCs. The proportions of each subcategory are presented in the figure legends.

To investigate the diversity and distribution patterns of BGCs in *Rhodococcus*, we retrieved and submitted for bioinformatics analysis all 616 *Rhodococcus* genomes from the NCBI genome database that passed quality filtering. In total, 48% of these genomes have been classified into 37 *Rhodococcus* species levels, while the species information of the other genomes has not been accurately identified. Therefore, the average nucleotide identity (ANI) of pairwise genomes was calculated and used to classify them into different phylogenomic-clades. By optimizing the ANI threshold, we found that at a value of 90, most different *Rhodococcus* species could be classified into distinct phylogenomic clades. At this threshold, the majority of genomes (597 out of 616) were grouped into 29 clades, while the remaining 19 genomes existed as singletons. Subsequently, the hierarchical dendrogram of these genomes was constructed based on the pairwise ANI values, and the genomic features and the number of different classes of BGCs in these genomes were also displayed on the outer ring of the dendrogram. As shown in Figure 1 and Table S1, genomes from different phylogenomic clades are mostly clustered together, and the top ten largest clades contain 494 genomes, accounting for 80.2% of all genomes. Among them, 131 genomes are completely assembled, 257 genomes are assembled at the scaffold level, and 228 genomes are assembled at the contig level. Despite originating from the same genus, these *Rhodococcus* genomes exhibit significant size variation, ranging from approximately 3.7 Mb to 11.7 Mb. For instance, genomes within clade 6 exhibit larger genome sizes, with nearly all surpassing 8 Mb in clade 6, while genomes within clade 4 have smaller genome sizes, generally less than 5 Mb. Additionally, the completeness of the *Rhodococcus* genomes is relatively high, with 85% containing fewer than 100 contigs, indicating a substantial degree of assembly quality.

All the 616 genomes yield a total of 12,455 BGCs, with lengths ranging from 1.0 to 183.6 kb, resulting in an average of 20 BGCs per *Rhodococcus* genome. The top three dominant classes of BGCs in *Rhodococcus* are NRPS (5374, 43.1%), RiPPs (1251, 10.0%), and Terpene (1156, 9.3%). It is worth noting that the average genome size of clade 6 is significantly larger than that of other clades (9.0 vs. 6.0 Mb), resulting in a notably higher number of BGCs encoded compared to other clades (31.6 vs. 20.2). Interestingly, *Rhodococcus* exhibits significant clade-specificity in gene cluster composition. For instance, the number of terpene gene clusters encoded by strains in clade 1 is significantly lower than those in clade 2 and 3, while the number of RiPP gene clusters is notably higher. Furthermore, genomes in clades 4 contain a higher abundance of PKSother BGCs compared to other clades, whereas genomes in clade 8 stand out for their possession of PKS-NRP_hybrid BGCs.

### 2.2. The Distribution Pattern of GCFs in Rhodococcus

**Figure 2:**
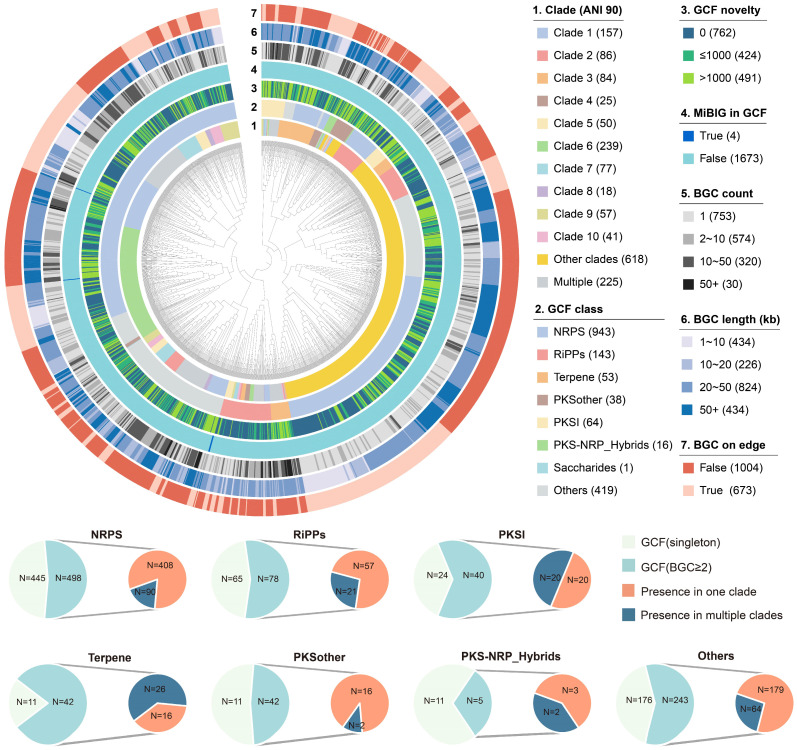
Hierarchical clustering of the 1677 GCFs in *Rhodococcus* genomes. Representation of the data (inner to outer layers): phylogenomic clade, GCF class, the novelty of GCFs, GCF containing BGCs from MiBIG or not, and BGC counts in each GCF. The outer two layers indicate the length and completeness of the representative BGC in each GCF. The proportions of each subcategory of GCFs are presented in the figure legends.

To accurately assess the specificity of secondary biosynthetic potential in *Rhodococcus*, all the predicted 12,455 BGCs were organized into a gene cluster network comprising 1677 GCFs at the established threshold of 0.3 using BIG-SCAPE. The hierarchical dendrogram of the resulting 1677 GCFs is presented in Figure 2 and Table S2, revealing that 86.5% of GCFs are only distributed in a specific phylogenomic clade of *Rhodococcus*, with the most predominant types of GCFs being NRPS and RiPPs. In total, 762 of these GCFs (45.4%) showed an average cumulative BLAST score of 0 to characterized BGCs from MiBIG and only 491 of GCFs (29.3%) showed an average cumulative BLAST score larger than 1000. In addition, only five GCFs contain BGCs from MiBIG, further implying the novelty of the GCFs encoded by *Rhodococcus*. The network analysis reveals that the GCFs containing BGCs responsible for synthesizing heterobactins, corynecins, rhodochelins, and aurachins include 9, 19, 32, and 4 gene clusters, respectively [13,18,19,20]. In addition to these, several GCFs exhibit a high degree of similarity to BGCs known to encode compounds such as ectoine and erythrochelin, indicating the potential for the production of similar compounds. Despite including all 616 genomes data from the same genus in this study, only 30 GCFs contained more than 50 BGCs, while 320 GCFs contained between 10 and 50 BGCs. These findings indicate significant diversity and variation in BGCs encoded by *Rhodococcus* species across different strains. The average length of BGCs in about 88% of GCFs was greater than 10 kb, with 25.9% being longer than 50 kb. Additionally, the shorter gene clusters were primarily found in singleton GCFs, indicating that GCFs containing two or more gene clusters had relatively higher completeness and quality. Notably, 40.1% (673 out of 1677) of the representative BGCs were located at the edge of the corresponding contigs and were mainly observed in NRPS GCFs, demonstrating that the fragmentation of genome sequences has significant implications for the mining of NRPS GCFs. Further analysis revealed that the proportion of clade-specific GCFs among the seven categories ranged from 38% to 89%. Specifically, the PKSother category had a higher proportion of clade-specific GCFs at 89%. These findings provide an important reference value for studying the evolutionary patterns of *Rhodococcus* and its secondary metabolic functions.

**Figure 3:**
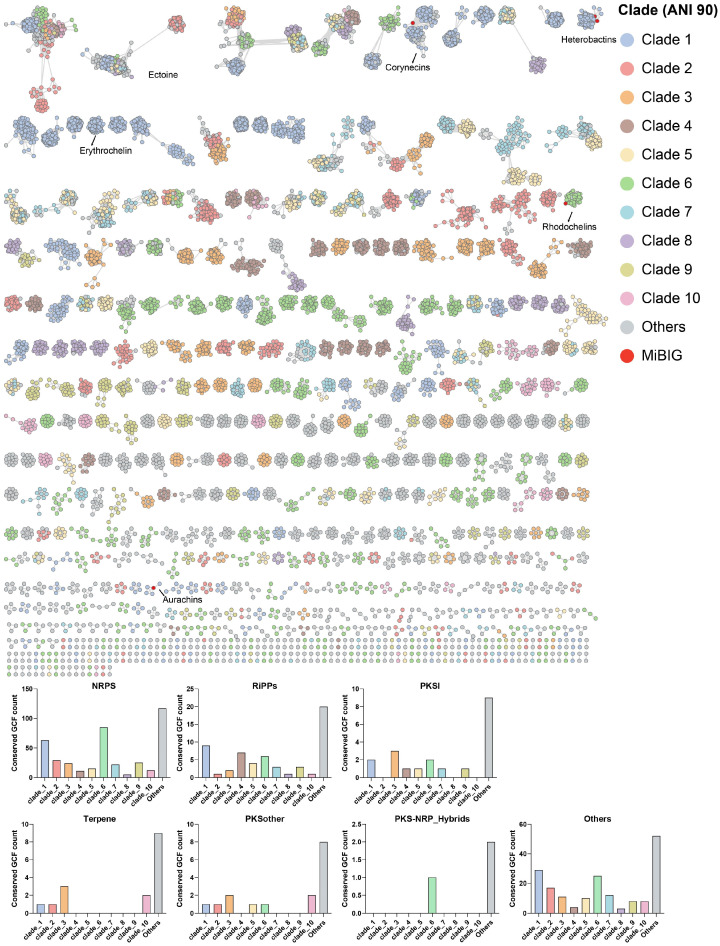
Sequence similarity network of 12,455 BGCs (743 singletons not shown) predicted from 616 *Rhodococcus* genomes. Each node represents one BGC, connected by edges when sharing a raw distance ≤0.3. The network is organized by different categories and the BGCs are colored according to the phylogenomic clade of its source genome.

The diversity and specificity of BGCs in *Rhodococcus* were visually demonstrated through the BIG-SCAPE sequence similarity network under mix mode (Figure 3). The NRPS category comprised 53.9% (498) of the total GCFs (≥2 BGCs) in the network, followed by 26.3% (243) others and 8.4% (78) of RiPP GCFs. The distribution of GCF in the top ten phylogenomic clades can be visually distinguished from the network, and the numbers of clade-specific GCFs of different types are also summarized. The results indicate that there is a significant difference in the number of clade-specific GCF encoded by the genomes in these seven categories of GCF. Among them, clades 6 and 1 have the highest number of clade-specific GCFs, mainly distributed in NRPS, others, and RiPP classes of GCFs. In the top ten clades, three lack clade-specific PKSI class GCFs, while six do not encode clade-specific terpene class GCFs, highlighting substantial evolutionary rate disparities among these GCFs.

### 2.3. Clade Specificity of Rhodococcus in RiPP Biosynthesis

**Figure 4:**
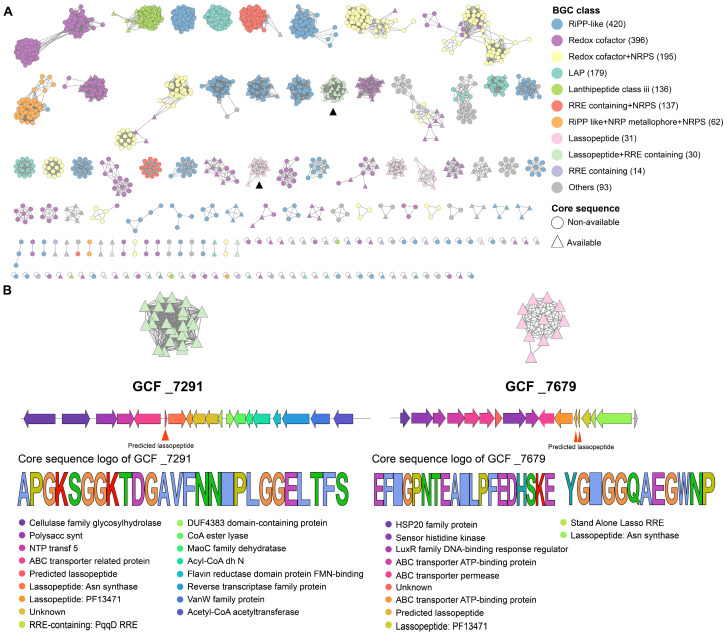
(**A**) A sequence similarity network was constructed for 1251 RiPP gene clusters predicted from 616 *Rhodococcus* genomes. The network also included 442 gene clusters categorized as ‘Others’ as they exhibit distances below the threshold to RiPP gene clusters. The subclasses of these biosynthetic gene clusters (BGCs) were annotated using the analysis results from antiSMASH, along with the annotation of BGCs predicted with core peptides using DeepRiPP. (**B**) BGC architecture and core peptide sequence logo of two clade-specific gene cluster families (GCF_7291 and GCF_7679).

Given that *Rhodococcus* encodes a large number of RiPP gene clusters, which are important sources of antimicrobial peptides, this study further analyzed the diversity and clade-specificity of *Rhodococcus* in RiPP biosynthesis. As shown in Figure 4A, the RiPP category gene clusters defined by BiG-SCAPE exhibit significant diversity and can be classified into subcategories such as RiPP-like (420), Redox cofactor (396), LAP (179), lanthipeptide-class-III (136), lassopeptide (31), and RRE containing (14), with the dominant subcategory being RiPP-like (24.8%). The core peptides of all the 1251 RiPP category gene clusters were predicted by antiSMASH or DeepRiPP, leading to the discovery of 891 core peptides from 459 BGCs, which mainly come from categories such as lanthipeptide-class-III (130), Redox cofactor (117), and RiPP-like (92), lassopeptide (31), LAP (17), RRE containing (11). It is worth noting that all the 460 RiPP gene clusters with predictable core peptides are distributed among 90 GCFs, with 78 being clade-specific, such as GCF_7291 and GCF_7679, which are only observed in *Rhodococcus* genomes of clade 4 (Figure 4B). Among the 78 clade-specific GCFs, 30 of them contain more than two BGCs, with the largest GCF containing 120 lassopeptide gene clusters that are solely distributed in clade 1. All 26 gene clusters in GCF_7291 encoded a single lassopeptide, with the core peptide predicted as APGKSGGKTDGAVFNNIPLGGELTFS. This core peptide exhibited only 42.3% sequence identity to predicted lasso peptides from antiSMASH-DB 3.0, demonstrating the high potential to be a novel lasso peptide. All the 11 gene clusters in GCF_7679 encoded two lassopeptides, with the core peptide predicted as EFIGPNTEAILPFEDHSKE and YGIGGQAEGWNP, respectively. In addition, this gene cluster is probably capable of producing two macrolactams, EFIGPNTE and YGIGGQAE. Both of these two GCFs encode the ABC transporter, asparagine synthetase, and transglutaminase (PF13471), which are essential for lassopeptide biosynthesis. The specific distribution of these gene clusters suggests that *Rhodococcus* strains at similar evolutionary statuses may use different natural weapons for environmental adaptation or defense. To further investigate these core peptides, we utilized two recently reported deep-learning algorithms to predict their activity. These algorithms combine multiple natural language processing neural network models, including LSTM, attention mechanisms, BERT, and XGBoost [21,22]. Consequently, we identified thirteen potential antimicrobial peptides from these core peptides (Table S3), whose antimicrobial activity merits further research and validation.

### 2.4. Secondary Metabolites of a Marine-Derived Rhodococcus Isolate

**Figure 5:**
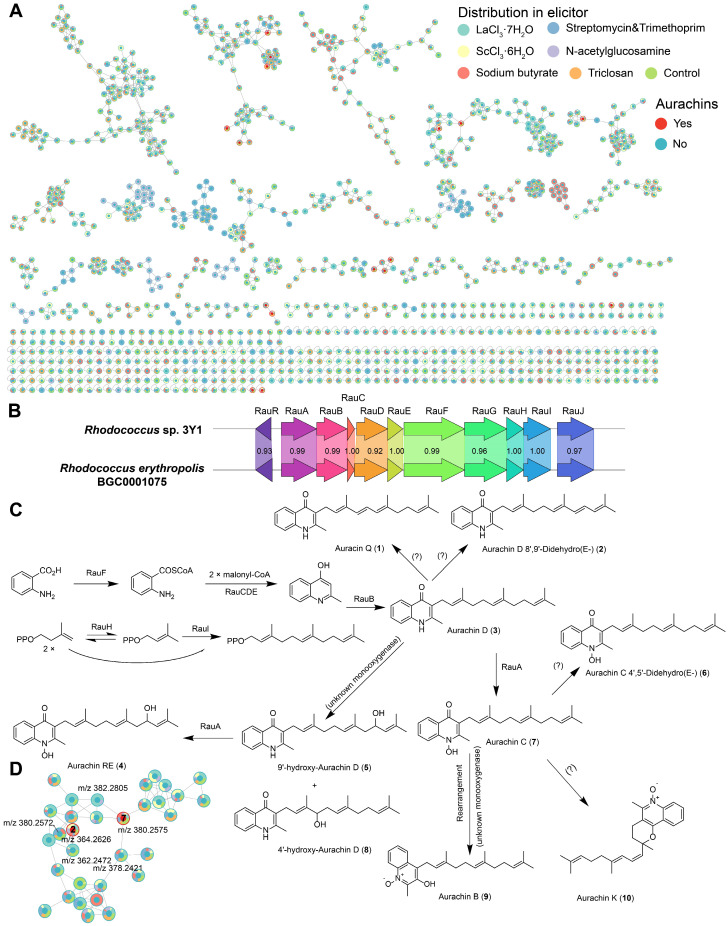
(**A**) Molecular network of crude extracts of *Rhodococcus* sp. 3Y1 cultured under different culture conditions. The pie chart colors on the nodes represent the distribution across different elicitor groups. Node color reflects whether the metabolites could be annotated as Aurachins. (**B**) The putative biosynthetic gene cluster responsible for the biosynthesis of aurachins. (**C**) Chemical structures and biosynthetic pathways of Aurachins, and “?” indicates unknown enzyme. (**D**) A molecular family annotated as Aurachins.

Strain 3Y1 was isolated from seawater at a depth of 3000 m in the Massau Trench in the Pacific Ocean at coordinates 148°53.3246′ E, 00°53.8546′ N. The 16S rRNA gene sequence of strain 3Y1 was found to be identical to that of *Rhodococcus qingshengii* JCM 15477^T^ [23]. According to Lee and Kim [24], this species is a later heterotypic synonym of *Rhodococcus erythropolis*. Therefore, the strain was identified as *Rhodococcus* sp. 3Y1. Secondary metabolites of this strain cultivated under seven different culture media in the presence or absence of six chemical elicitors were comprehensively analyzed using high-resolution liquid chromatography-mass spectrometry. The resulting mass spectra were analyzed using the Global Natural Products Social Molecular Networking (GNPS, https://gnps.ucsd.edu/, accessed on 15 May 2024) workflow, with annotation performed through the Feature-Based Molecular Networking (FBMN) workflow and Dereplicator+. Chemical features detected in the blank samples (culture media) were excluded from the analysis. Using FBMN analysis, a molecular network comprising 1139 features and 451 molecular families was generated. As showed in Figure 5A and Table S4, Dereplicator+ identified a series of aurachin-like compounds. Due to the high structural similarity of these compounds, it was challenging to accurately identify them using solely in silico MS/MS methods. Therefore, unreliable matches were discarded, and manual comparisons were performed to preliminarily identify certain compounds. Additionally, over 90% of the features remained uncharacterized, indicating the presence of potentially novel compounds in *Rhodococcus* that warrant further investigation.

We utilized six different elicitors with various induction mechanisms [25]: lanthanum chloride and scandium chloride as rare metals, N-acetylglucosamine as a oligosaccharides source, sodium butyrate as an HDAC inhibitor, and three antibiotics as competitive agents. Interestingly, under all elicitor conditions, we discovered that the strain *Rhodococcus* sp. 3Y1 produced a series of aurachin-like compounds: Aurachin Q (**1**), Aurachin D 8′,9′-Didehydro (**2**), Aurachin D (**3**), Aurachin RE (**4**), 9′-hydroxy-Aurachin D (**5**), Aurachin C 4′,5′-Didehydro (**6**), Aurachin C (**7**), 4′-hydroxy-Aurachin D (**8**), Aurachin B (**9**) and Aurachin K (**10**). Figure 5D showed a molecular family of aurachin-like compounds, comprising a total of 32 features. Among these, two features have been identified as aurachin-like compounds. One feature was identified as Aurachin C, and another feature was identified as Aurachin D 8′,9′-Didehydro. Several features directly connected to the identified ones may be novel analogs (probably hydrogenation or dehydrogenation) of these identified aurachins. Aurachins and related compounds have been recognized as potent inhibitors of mitochondrial respiration, primarily by blocking NADH oxidation. This inhibitory action is likely attributed to their structural similarity to vitamin K [26]. Aurachin RE, a prenylated quinoline antibiotic first isolated from the genus *Rhodococcus* [27], is biosynthesized by BGC0001075 [19]. Using antiSMASH analysis, we successfully identified the biosynthetic gene cluster responsible for producing aurachins in *Rhodococcus* sp. 3Y1. This BGC shows a high degree of similarity to the previously reported BGC0001075 (Figure 5B). As depicted in Figure 5C, the biosynthetic pathways for compounds **2**, **3**, **4**, **5**, and **6** are relatively well understood and reported [19,28,29]. However, the biosynthetic pathways for compounds **1** and **7** have not yet been clearly identified. Further investigation and exploration into the biosynthetic pathways of these compounds are warranted. Furthermore, under the conditions of sodium butyrate and N-acetylglucosamine, several unique molecular families were detected (Figure 5A). These molecular families have not been annotated, suggesting they may represent novel compounds.

## 3. Discussion

*Rhodococcus* is a versatile genus of Gram-stain-positive bacteria known for its remarkable metabolic diversity and ability to thrive in various environments, including soil, water, and even extreme habitats like deep-sea sediments [13]. This genus is of significant interest in the field of natural products due to its extensive secondary metabolite production, which includes antibiotics and other bioactive compounds. With the continuous accumulation of *Rhodococcus* strain resources and microbial natural products, efficiently discovering novel and highly active natural products from *Rhodococcus* has become a significant challenge. This study aims to analyze the diversity and specificity of the secondary biosynthetic potential of *Rhodococcus* from a phylogenomic similarity perspective rather than at the species level, with a particular focus on the potential for synthesizing phylogenomic clade-specific antimicrobial peptides from RiPP gene clusters. Using a deep-sea-derived *Rhodococcus* strain as an example, this study illustrates the discovery of unique and potential novel natural products using LC-MS and HiTES strategies.

Increasing evidence suggests that closely related strains can encode significantly different biosynthetic gene clusters or exhibit substantial differences in the composition of these biosynthetic gene clusters [12,13,14,15]. Two recent studies have reported the phylogenomic-dependent patterns of NRPS gene clusters in 30 and 110 *Rhodococcus* genomes, respectively, especially for BGCs predicted to encode the biosynthesis of lipopeptides [12,13]. The present study is the first to systematically and comprehensively analyze the distribution specificity of all eight classes of BGCs in 616 *Rhodococcus* genomes of 48 different phylogenomic-clades. The *Rhodococcus* genome dataset used in this study is about six times larger than those in previous studies. It also provides a detailed revelation of the clade-specific BGCs in the top ten phylogenomic clades of *Rhodococcus*, revealing that *Rhodococcus* may possess various chemical weapons for environmental adaptation or survival maintenance. These findings provide important clues for the targeted discovery of specific types of natural products and offer significant reference value for studying the genetic evolution and metabolic adaptability of *Rhodococcus*.

The quality of genome assembly and the algorithms used for BGC detection significantly impact the accuracy of identifying BGCs. Fragmented genome assemblies can result in the dispersion of genes from the same BGC across different contigs, potentially leading to an overestimation of BGC numbers. Conversely, if a BGC is excessively fragmented, prediction tools may fail to detect it, leading to an underestimation. In recent years, advancements in sequencing and bioinformatics technologies have substantially improved the accuracy and speed of BGC prediction in microorganisms. The most commonly used algorithms for BGC detection involve BLAST and Hidden Markov Model (HMM) comparisons. For instance, the widely utilized tool, antiSMASH [30], employs these methods to efficiently identify BGCs homologous to known clusters through database searches. We reanalyzed the complete genome of the first *Rhodococcus* strain, *Rhodococcus jostii* RHA1 [11], using the latest antiSMASH 7 tool. Our analysis identified 16 NRPS and 2 PKS BGCs, which contrasts with the previously reported 24 NRPS and 7 PKS BGCs. This discrepancy highlights the improvements in tools like antiSMASH, which have greatly enhanced the efficiency and accuracy of BGC prediction, particularly in high-quality genome assemblies. The combination of genome mining techniques and deep-learning algorithms has enabled the targeted discovery of numerous novel and highly active antimicrobial peptides [21,22]. In this study, deep-learning algorithms were used to rapidly predict 13 novel potential antimicrobial peptides from core peptides encoded by RiPP gene clusters of *Rhodococcus*. The activity of these putative antimicrobial peptides awaits experimental validation in future studies. Additionally, the post-translational modification processes of these core peptides may significantly impact the structure and activity of the final metabolites. Therefore, accurate prediction of the products encoded by specific RiPP gene clusters must consider the effects of these modifications.

In this study, we utilized a deep-sea-derived *Rhodococcus* sp. 3Y1 as an example to explore clade-specific natural products through the combination of LC-MS-based untargeted metabolomics with HiTES strategies. We identified a series of aurachin-like compounds and predicted their biosynthetic pathways through genome mining. Aurachins (Aurachin A–L) were first isolated from the myxobacterium *Stigmatella aurantiaca* Sg a15 [28], but their complete biosynthetic pathways remain unclear. Aurachin RE, a prenylated quinoline antibiotic, was first isolated from the genus *Rhodococcus* and exhibits potent antibacterial activity against a variety of Gram-positive bacteria [19]. Further studies isolated two additional aurachin-like compounds (Aurachin Q and Aurachin R) from *Rhodococcus* strains [7]. Aurachin R showed moderate antibacterial activity against *Staphylococcus epidermidis* DSM 20044, *Bacillus subtilis* DSM 347, and *Propionibacterium acnes* DSM 1897, while Aurachin Q did not exhibit antibacterial activity. The present study provides evidence for the targeted discovery of novel aurachin-like compounds, and highlights the structural diversity of aurachins.

## 4. Materials and Methods

### 4.1. Genome Collection and Phylogenomic Analysis

*Rhodococcus* genomes were downloaded from the NCBI web servers, with data up to date as of May 2024. After filtering for genomes with completeness greater than 90%, contamination less than 5%, and fewer than 200 contigs, 616 genomes were retained for further analysis. Assembly information and other relevant details were obtained and organized via FTP from NCBI. The average nucleotide identity between genomes was calculated using the pyANI package [31] to group phylogenomic-close genomes into clades, with an ANI threshold greater than 90%. Based on the ANI similarity matrix of all genomes, a dendrogram of these genomes was constructed using hierarchical clustering in R [32] and visualized with ITOL [33].

### 4.2. Diversity and Specificity of Biosynthetic Gene Clusters in Rhodococcus

Similar to our previous study [34], all genomes were processed using the command-line version of antiSMASH v7.0.1 with the bacterial setting and default parameters [30]. The number of each class of BGCs and relevant information regarding their architecture were extracted from the HTML files (antiSMASH outputs) using a customized Python toolkit (https://github.com/BioGavin/wlabkit, accessed on 15 May 2024). The diversity and novelty of all eight classes of BGCs were compared with known BGCs in the MiBIG v3.1 database using BIG-SCAPE at the default cut-off of 0.3 [35]. Each node in the network represents a BGC, and BGCs with similar Pfam domain units are connected by edges. Using the parameter -mix, the final analysis produced eight separate networks for each class of BGCs, as well as a mixed network combining all classes. All network was visualized using Cytoscape 3.10.2 [36]. The novelty of GCFs was assessed by calculating the average cumulative BLAST score against known BGCs in the MiBIG database using the antiSMASH function knownclusterblast [30]. For each of the 1677 GCFs, the BGC with the highest total BLAST score and the longest length was selected as the representative BGC. The class of the representative BGC, along with relevant information about them, was used for GCF clustering.

### 4.3. Diversity and Specificity of Rhodococcus in RiPP Biosynthesis

The putative core peptides of RiPP gene clusters were obtained using the software tools DeepRiPP v1.0.0 [37] and antiSMASH v7.0.1, and used to evaluate the diversity and specificity of *Rhodococcus* in RiPP biosynthesis. The predicted core peptide sequences of RiPP gene clusters in all *Rhodococcus* genomes were extracted from the DeepRiPP output and GBK files (antiSMASH outputs) using a customized Python toolkit (https://github.com/BioGavin/wlabkit, accessed on 15 May 2024). The sequence logo of predicted RiPP core sequence was constructed using TBtools [38]. This antimicrobial activity of the core peptides was predicted using two recently released deep-learning algorithms with the default settings [21,22].

### 4.4. High-Throughput Elicitor Screening (HiTES)

*Rhodococcus* sp. 3Y1 was isolated from a depth of 3000 m in the Massau Trench in the Pacific Ocean at coordinates 148°53.3246′ E, 00°53.8546′ N in January 2017. After sampling, 1 L of seawater was stored at 4 °C in a temperature-controlled cold storage. The strain was isolated using B1 isolation medium (0.5 g/L glucose, 0.05 g/L yeast extract, 0.1 g/L K_2_HPO4, 0.005 g/L MgSO_4_·7H_2_O, 3% sea salt, 1.5% agar, and 100 mg/L nystatin). The seawater samples were plated and incubated at 28 °C, and single colonies were obtained and stored in 40% glycerol at −80 °C. Genomic DNA was extracted using the Genomic DNA Mini Preparation Kit (Beyotime Institute of Biotechnology, Shanghai, China). The 16S rDNA gene sequence fragment of *Rhodococcus* sp. 3Y1 was amplified using polymerase chain reaction (PCR) with the primer 27F (5′-AGAGTTTGATCCTGGCTCAG-3′). The complete genome was subsequently obtained using a combination of third-generation sequencing with the Oxford Nanopore platform and second-generation sequencing with Illumina. To enhance the reliability of data processing, raw data from both the NovaSeq and GridION X5 platforms were first trimmed using Canu v1.8 [39] to produce high-quality clean reads. The paired-end Illumina reads from second-generation sequencing and the long reads from Nanopore were then assembled using Unicycler v0.4.5 [40], resulting in a high-quality complete genome assembly. The secondary metabolic potential of this strain was comprehensively investigated using a combined strategy of untargeted LC-MS-based metabolomic analysis and high-throughput elicitor screening. The HiTES experiments were conducted under 49 different laboratory culture conditions using seven distinct culture media: M1 (peptone 10 g, yeast extract 5 g, NaCl 10 g for 1 L; pH 7.2), M2 (peptone 5 g, yeast extract 1 g, Fe(III) citrate 0.1 g, NaCl 19.45 g, MgCl_2_ 5.9 g Na_2_SO_4_ 3.24 g, CaCl_2_ 1.8 g, KCl 0.55 g, NaHCO_3_ 0.16 g, KBr 0.08 g, SrCl_2_ 34 mg, H_3_BO_3_ 22 mg, Na-silicate 2.4 mg, NaF 2.4 mg, (NH_4_)NO_3_ 1.6 mg, Na_2_HPO_4_ 8 mg for 1 L; pH 7.2), M3 (yeast extract 0.5 g, glucose 2 g for 1 L; pH 5–6), M4 (tryptone 5 g, beef extract 3 g, NaCl 5 g for 1 L; pH 7.0), M5 (tryptone 2 g, yeast extract 1 g, glucose 2 g for 1 L; pH 5–6), M6 (peptone 0.5 g, yeast extract 0.5 g, casein peptone 0.5 g, glucose 0.5 g, Soluble amylose 0.5 g, KH_2_PO_4_ 0.3 g, MgSO_4_·7H_2_O 24 mg, sodium pyruvate 0.3 g for 1 L; pH 7.2), M7 (yeast extract 1 g, MgSO_4_·7H_2_O 0.2 g, NaCl 0.4 g, mannitol 10 g for 1 L; pH 7.4). The pre-culture was performed in medium 2216E for 3 days, after which the strain was inoculated into seven different liquid media (M1~M7). Fermentations were carried out in 12 mL cell culture tubes containing 6 mL of medium at 30 °C with shaking at 150 rpm for 3 days. Subsequently, six different elicitors—LaCl_3_·H_2_O (2 mM), ScCl_3_·6H_2_O (200 μM), N-acetylglucosamine (100 mM), sodium butyrate (100 mM), streptomycin with trimethoprim (33 μM), and triclosan (5 μM)—were added and the cultures were incubated for an additional 7 days. Blank and control groups were set up for subsequent analysis.

For the extraction of the liquid cultures, 6 mL of culture broth was ultrasonicated and extracted twice with an equal volume of ethyl acetate (EtOAc). The combined EtOAc layers were transferred to a 10 mL sample bottle and dried under vacuum. The resulting crude extracts were re-dissolved in 0.5 mL of methanol (MeOH) and transferred to 1.5 mL centrifuge tubes. These tubes were then concentrated using a termovap sample concentrator. The concentrated extracts were re-dissolved in 50 μL of MeOH and transferred to 1.5 mL vials. The dissolved extracts were centrifuged at 12,000 rpm for 10 min, and the supernatant was filtered through a 0.22 µm nylon syringe filter before injection.

### 4.5. UPLC-QTOF-MS/MS Analysis

Similar to our recent study [34], the crude extract was analyzed using a SCIEX X500B Q-TOF spectrometer coupled to an ExionLC AC system under the following LC conditions: 1–2 min (10% methanol in H_2_O), 2–18 min (10–100% methanol), 18–22 min (100% methanol), and 22.01–25 min (10% methanol) at a flow rate of 0.3 mL/min and a column temperature of 40 °C. The QTOF MS settings during the LC gradient were as follows: positive ion mode mass range 200–1500 *m*/*z*, total scan time 0.495 s, maximum candidate ions 5, and ion source temperature 600 °C. MS2 fragmentation was performed with a QTOF mass range of 50–1000 *m*/*z*, fixed collision energy of 30 V, fixed collision energy spread of 10 V, and an ion spray voltage of 5.5 kV.

The raw LC-MS data files were converted to mzML format using MSConvert software v3.0.24109 [41] and subsequently processed using MZmine2 software v2.53 [42]. Feature detection, isotope grouping, and alignment were performed following the feature-based molecular networking (FBMN) documentation [43]. The data were filtered by removing all MS/MS peaks from blank media. A CSV file and an MGF file were generated from MZmine2 and uploaded to the FBMN workflow in GNPS (http://gnps.ucsd.edu, accessed on 15 May 2024). Molecular networks were generated with default parameters. Additionally, the MGF file was uploaded to the Dereplicator+ workflow in GNPS (http://gnps.ucsd.edu, accessed on 15 May 2024) with default parameters [44]. The molecular network from FBMN was also visualized using Cytoscape 3.10.2.

## 5. Conclusions

In conclusion, the current study uncovered that 87.7% of GCFs are uniquely found in a specific phylogenomic clade of *Rhodococcus*, with NRPS and RiPPs being the most prevalent types of gene clusters. Through extensive genome mining and deep-learning analysis, it was revealed that *Rhodococcus* harbors a substantial number of clade-specific novel RiPPs, some of which could exhibit antibacterial properties. The HiTES investigation indicate that certain elicitors can stimulate a marine-derived *Rhodococcus* strain to produce a plethora of potentially new aurachin-like compounds. This study offers valuable insights for targeted exploration of novel natural products from *Rhodococcus*, particularly focusing on clade-specific metabolites.

## Data Availability

The whole genome sequence data reported in this paper have been deposited in the Genome Warehouse of the National Genomics Data Center, China National Center for Bioinformation, under the BioProject accession number PRJCA029818. Additionally, the data are also available in the NCBI under BioProject accession number PRJNA1155922.

## Supplementary Materials

The following supporting information can be downloaded at: https://www.mdpi.com/article/10.3390/md22090409/s1, Table S1: Genomic features and the antiSMASH results of the 616 *Rhodococcus* genomes; Table S2: Information about the 12,455 BGCs; Table S3: Information about the RiPPs, BGCs and core peptides; Table S4: Metabolite annotation in metabolomic data of *Rhodococcus* sp. 3Y1.
